# Нарушения функции гипофиза у пациентов с терминальной стадией хронической почечной недостаточности

**DOI:** 10.14341/probl13212

**Published:** 2024-01-24

**Authors:** Т. Н. Маркова, Е. В. Косова, Н. К. Мищенко

**Affiliations:** Московский государственный медико-стоматологический университет им. А.И. Евдокимова; Городская клиническая больница № 52 ДЗМ; Московский государственный медико-стоматологический университет им. А.И. Евдокимова; ООО «Мед Гарант»

**Keywords:** гипофиз, гормоны, хроническая болезнь почек, гемодиализ, перитонеальный диализ, трансплантация почки

## Abstract

Патологические изменения в работе почек приводят к нарушению поддержания гомеостаза внутренней среды организма. По мере снижения скорости клубочковой фильтрации и нарастания уремии уменьшается метаболизм, изменяются процессы транспортировки и связывания с клетками-мишенями многочисленных биологически активных веществ, в том числе гормонов гипофиза. В статье представлен обзор нарушений функции гипофиза у пациентов с терминальной стадией хронической болезни почек (тХБП) и обсуждены патогенетические механизмы их формирования. Особое внимание уделено оценке изменений концентрации гормонов гипофиза у пациентов, получающих заместительную почечную терапию (ЗПТ). Так, тХБП приводит к повышению уровня пролактина, лютеинизирующего гормона (ЛГ) и фолликулостимулирующего гормона (ФСГ). Концентрации соматотропного гормона (СТГ), инсулиноподобного фактора роста-1 (ИФР-1), тиреотропного гормона (ТТГ), адренокортикотропного гормона (АКТГ) и вазопрессина могут оставаться в пределах нормальных значений или повышаться в данной группе больных. Проведение ЗПТ не снижает уровни пролактина, ЛГ, ФСГ, в то же время концентрации СТГ, ИФР-1, ТТГ имеют тенденцию к нормализации. Содержание АКТГ и вазопрессина может оставаться без изменений или уменьшаться. Трансплантация почки в большинстве случаев корректирует описанные нарушения. В целом устранение гормональных изменений способно улучшить клинический исход и качество жизни пациентов, страдающих тХБП.

## Abstract (en)

Disorders in the kidneys lead to disturbance of homeostasis. As the glomerular filtration rate decreases, the metabolism of numerous biologically active substances, including pituitary hormones, decreases. The article presents an overview of pituitary dysfunction in patients with chronic kidney disease (CKD) and discusses the possible reasons of the pathogenetic mechanisms. Particular focus is being given to the assessment of changes in the concentration of pituitary hormones in patients with end-stage chronic kidney disease (CKD) and discusses the pathogenetic mechanisms of their formation. Particular attention is paid to the assessment of changes in the concentration of pituitary hormones in patients receiving renal replacement therapy (RRT). CKD leads to an increase in the level of prolactin, luteinizing hormone (LH) and follicle-stimulating hormone (FSH). Concentrations of growth hormone (GH), isulin-like growth factor-1 (IGF-1), thyroid-stimulating hormone (TSH), adrenocorticotropic hormone (ACTH) and vasopressin may remain within normal values or increase in this group of patients. RRT does not reduce the levels of prolactin, LH, FSH, while the concentration of growth hormone, IGF-1, TSH tends to normalize. The content of ACTH and vasopressin may remain unchanged or decrease. Kidney transplantation in most cases corrects hormonal disorders. Correction of hormonal changes can improve the clinical outcome and quality of life of patients with end stage CKD.

## МЕТОДОЛОГИЯ ПОИСКА ИСТОЧНИКОВ

В процессе написания статьи использовались следующие базы данных: www.elibrary.ru, www.ncbi.nlm.nih.gov/pubmed, www.clinicalTrials.gov, поисковая система Google. Поиск проводился по ключевым словам: гипофиз, гормоны, хроническая болезнь почек, гемодиализ, перитонеальный диализ, трансплантация почки.

## ВВЕДЕНИЕ

В последние десятилетия хроническая болезнь почек (ХБП) стала глобальной проблемой системы здравоохранения наряду с другими хроническими неинфекционными заболеваниями. Это связано как с ростом заболеваемости ХБП, так и с высокой смертностью пациентов, получающих заместительную почечную терапию (ЗПТ), главным ­образом, вследствие развития патологии сердечно-сосудистой системы [1]. По результатам крупных когортных исследований, общемировая распространенность ХБП составляет в среднем от 11 до 13% [2]. Кроме того, к 2040 г., по прогнозам экспертов, данная патология станет пятой причиной смерти населения во всем мире [3]. К основным нозологиям, вызывающим ХБП, сегодня относят сахарный диабет, гипертоническую болезнь, хронический гломерулонефрит, а также сочетания данных заболеваний [4].

Патологические изменения в работе почек приводят к нарушению гомеостаза внутренней среды организма. При наличии терминальной стадии ХБП (тХБП) нарушаются метаболизм и транспортировка гормонов, а также снижается чувствительность клеток-мишеней [5]. Данные изменения требуют более тщательного контроля у пациентов с тХБП, получающих ЗПТ.

## ГОРМОН РОСТА (СОМАТОТРОПИН)

Гормон роста (ГР), или соматотропин, представляет собой полипептид, состоящий из 191 аминокислоты [6]. ГР связывается со специфическим рецептором преимущественно в печени, а также в других органах, например в почках, и стимулирует секрецию инсулиноподобного фактора роста-1 (ИФР-1) [6][7]. В целом метаболические эффекты ГР можно рассматривать как комбинированное действие ГР и ИФР-1 [6].

Особый интерес представляет влияние ГР и ИФР-1 на строение и функцию почек. Система ГР/ИФР-1 играет ключевую роль в нормальном развитии почек и регуляции клубочковой гемодинамики вследствие расширения приносящих и выносящих артериол клубочка, а также стимуляции реабсорбции фосфатов (Рi), натрия (Na+) и воды в канальцах почек. ИФР-1 усиливает синтез кальцитриола с последующим увеличением реабсорбции кальция (Cа2+). Система ГР/ИФР-1 повышает выделение аммиака (NH4+) с мочой и увеличивает глюконеогенез, протекающий в клетках проксимальных канальцев [7]. Влияние ГР и ИФР-1 на почки представлено на рисунке 1.

**Figure:**
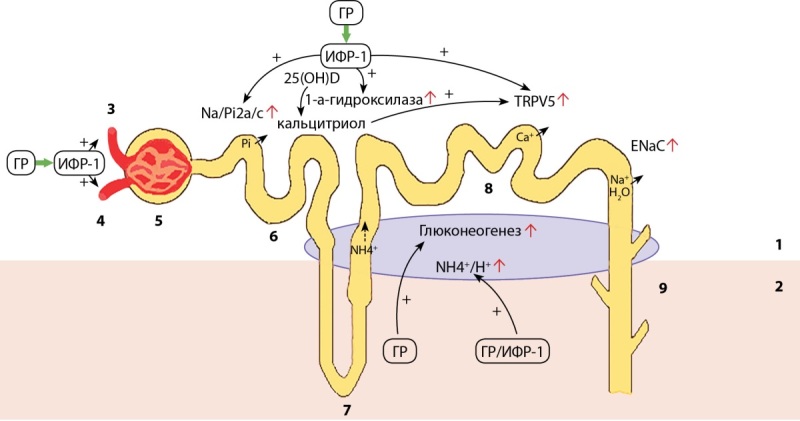
Рисунок 1. Влияние ГР и ИФР-1 на почки [адаптировано из 7].1 — корковое вещество; 2 — мозговое вещество; 3 — приносящая артериола; 4 — выносящая артериола; 5 — клубочек; 6 — проксимальный извитой каналец; 7 — петля Генле; 8 — дистальный извитой каналец; 9 — собирательная трубочка.Примечание: ГР — гормон роста; ИФР-1 — инсулиноподобный фактор роста-1; Na+ — натрий; Н2О — вода; Na-Pi2a/с — натрий-фосфорный канал; Pi — фосфор; 25(ОН)D-25 — гидроксивитамин D; TRPV5 — кальциевый канал; ENaC — эпителиальный натриевый канал; NH4+ — аммиак; Н+ — водород.

При развитии тХБП концентрация ГР в сыворотке крови не изменена или незначительно повышена. Повышение уровня ГР связано с нарушением почечного клиренса и развитием резистентности к ГР. Резистентность к данному гормону обусловлена снижением плотности рецепторов в различных клетках-мишенях, а также развитием пострецепторных дефектов при передаче сигналов ГР и уменьшением концентрации свободного ИФР-1 на стадии ХБП5 [8]. Уменьшение содержания данного вещества вызвано повышением уровней ИФР-связывающих белков (ИФРСБ) в связи с нарушением их выведения вследствие развития ХБП [8].

Также было показано, что при прогрессирующей почечной недостаточности развивается метаболический ацидоз. Данное патологическое состояние может снизить экспрессию рецепторов к ГР и уменьшить синтез ИФР-1 печенью [9]. Описанные выше изменения в гомео­стазе гормонов системы ГР/ИФР-1 способны уменьшить скорость клубочковой фильтрации (СКФ), а также вызвать повреждения подоцитов. Влияние данных гормонов на баланс воды и электролитов при тХБП до конца не определено, так как эффекты ГР носят не только самостоятельный характер, но и опосредованы действием ИФР-1 [9]. Таким образом, тХБП можно охарактеризовать как состояние, при котором изменяются регуляция и биодоступность компонентов системы ГР/ИФР-1, приводящее к снижению активности ГР и ИФР-1.

Концентрация ГР и ИФР-1 у пациентов с тХБП, получающих ЗПТ, изменяется в зависимости от выбора тактики лечения. Так, после начала диализной терапии уменьшается уровень ГР и повышается содержание ИФР-1 в сыворотке крови, что, вероятно, вызвано удалением из кровеносного русла ИФР-связывающих белков. После трансплантации почки происходит нормализация метаболизма гормонов системы ГР/ИФР-1 [5].

Особое клиническое значение имеет применение ГР в практике лечения больных, получающих ЗПТ. У таких пациентов возможно развитие белково-энергетической недостаточности (БЭН), неблагоприятно влияющей на исходы. Одной из терапевтических стратегий лечения БЭН является назначение рекомбинантного ГР (рГР) [10]. Применение рГР в группе пациентов, получающих терапию гемодиализом и имеющих уровень альбумина <4 г/дл, продемонстрировано в крупном многоцентровом рандомизированном исследовании, оценивающем эффективность данного вещества по сравнению с плацебо. Назначение рГР на протяжении 20 нед в описанной группе больных приводило к снижению индекса массы тела и общего содержания жировой ткани, уменьшению уровня С-реактивного белка и гомоцистеина наряду с повышением уровня холестерина липопротеинов высокой плотности и трансферрина в сыворотке крови по сравнению с группой плацебо. В то же время общая смертность, смертность от сердечно-сосудистых заболеваний, концентрация альбумина в сыворотке крови, содержание мышечной массы, толерантность к физической нагрузке и качество жизни пациентов достоверно не различались в двух группах, что может быть связано с небольшой продолжительностью исследования и ограниченными размерами выборки. Таким образом, применение рГР у пациентов, получающих терапию гемодиализом, снизило факторы риска развития сердечно-сосудистых заболеваний. В то же время не было получено достоверного улучшения белково-энергетического баланса [11]. Дальнейшие исследования помогут определить четкие клинические рекомендации по тактике назначения рГР в группе больных, страдающих тХПН, для улучшения качества жизни и снижения смертности от сердечно-сосудистых заболеваний.

## ПРОЛАКТИН

Пролактин — это полипептидный гормон, состоящий из 199 аминокислот [12]. Данный гормон оказывает влияние на репродуктивную систему как у женщин, так и у мужчин, регулируя уровни гонадотропинов [12][13]. Однако, наряду с влиянием на половую систему, пролактин участвует в поддержании водно-солевого обмена, поскольку обладает антидиуретическим эффектом [14]. По данным Брин В.В. и соавт. (2001), пролактин вызывает увеличение реабсорбции воды, снижение экскреции Na+ и повышение экскреции K+, Pi, магния (Мg2+) и Ca2+ с мочой [15]. Исследователи Rojas-Vega L. и соавт. (2015) показали, что пролактин приводит к активации натрий-хлоридного симпортера, который стимулирует реабсорбцию Na+ и хлора (Cl-) в дистальном извитом канальце почек [16]. Также пролактин стимулирует синтез кальцитриола в клетках проксимальных канальцев почек [14]. Таким образом, данный гормон имеет широкое биологическое действие в отношении электролитного обмена. У пациентов с тХБП уровень пролактина повышен как в период до начала терапии ЗПТ, так и на фоне терапии гемодиализом или перитонеальным диализом. Гиперпролактинемия в данной группе больных выявляется у 70–90% женщин и у 20–50% мужчин [17]. Одновременно с ухудшением функции почек уровень пролактина увеличивается, достигая высоких значений у пациентов, получающих ЗПТ [5]. Так, по данным исследования Lo J.C. и соавт. (2017), медиана концентрации пролактина в сыворотке крови пациентов, получающих терапию гемодиализом, составила 65 нг/мл. Повышение уровня данного гормона наблюдалось вне зависимости от частоты проведения ЗПТ [18].

Среди основных причин гиперпролактинемии при уремическом состоянии выделяют снижение почечного клиренса пролактина [19] и увеличение продукции пролактина вследствие сниженного синтеза дофамина [20].

Также считается, что гиперпролактинемия может являться компенсаторным механизмом, развивающимся вследствие гипокальциемии, поскольку пролактин является одним из факторов, стимулирующих синтез кальцитриола [21].

В настоящее время известно, что данный гормон участвует в развитии атеросклероза и эндотелиальной дисфункции [22]. Так, в когортном исследовании, проведенном Carrero J. и соавт. (2012), сообщалось, что повышение концентрации пролактина в сыворотке крови на каждые 10 нг/мл увеличивает как риск сердечно-сосудистых событий у пациентов с ХБП, не получающих лечение диализом, так и смертность вследствие сердечно-сосудистых заболеваний среди пациентов, получающих ЗПТ [23]. По данным Марковой Т.Н. и соавт. (2019), гиперпролактинемия встречается у 39,8% пациентов, имеющих ХБП С3б-С5. В исследовании женщины с ХБП имели уровень общего пролактина (1065 [ 694; 1818] мМЕ/л) почти вдвое выше, чем у мужчин (523 [ 428; 635] мМЕ/л; pm-u=0,03), аналогичная закономерность выявлена при изучении содержания мономерного пролактина: 857 [ 605; 1150] мМЕ/л против 449 [ 406; 588] мМЕ/л соответственно (pm-u=0,008). Также показано, что диализные методы лечения в 2,5 раза увеличивают частоту гиперпролактинемии вне зависимости от проводимого вида ЗПТ (перитонеальный диализ или гемодиализ) в сравнении с пациентами с ХБП 3б стадии и выше. У лиц на диализной терапии увеличение концентрации пролактина выявлено в 47,8%, в то время как у додиализных пациентов — в 19,2% случаев (рχ²=0,012) [24]. Важно отметить, что ЗПТ не приводит к нормализации концентрации пролактина [22][24], в то же время трансплантация почки с последующим улучшением клубочковой фильтрации снижает содержание данного гормона в сыворотке крови до нормальных значений. В исследовании Kumar R. и соавт. (2014) выявлено, что содержание пролактина уменьшилось через 6 мес после трансплантации почки, однако при динамическом наблюдении нарушение функции трансплантата вновь вызывало повышение уровня данного гормона [25].

В настоящее время четкие рекомендации по тактике диагностики и лечения гиперпролактинемии у пациентов, получающих ЗПТ, не разработаны. Однако предполагается, что показанием для назначения медикаментозной терапии является наличие нарушений менструального цикла у женщин репродуктивного возраста, имеющих тХБП [5]. В целом гиперпролактинемия, выявляемая у пациентов с ХБП, требует дальнейшего изучения, определения тактики лечения и прогноза.

## ГОНАДОТРОПИНЫ

К гонадотропинам относят гликопротеины — лютеинизирующий гормон (ЛГ) и фолликулостимулирующий гормон (ФСГ). Высвобождение данных веществ в кровеносное русло стимулируется гонадотропин-рилизинг-гормоном (ГнРГ) [26][27].

При тХБП происходит снижение амплитуды выработки ГнРГ в гипоталамусе, что вызывает нарушение пульсовой секреции ЛГ как у мужчин, так и у женщин [28]. Данные нарушения могут быть вызваны гиперпролактинемией. Также повышенная концентрация ЛГ вследствие измененного почечного клиренса гормонов может снижать выработку ГнРГ [28–30]. При уремии происходит снижение уровня тестостерона у мужчин, что способствует повышению концентрации ЛГ [29][30]. Женщины с тХБП имеют нарушения циклического высвобождения ЛГ, которые приводят к отсутствию наступления овуляции и являются частой причиной развития бесплодия [28]. Таким образом, большинство пациентов с тХБП имеют повышенный базальный уровень ЛГ [28–30].

Концентрация ФСГ у мужчин с уремическим синдромом также может быть увеличена, как и уровень ЛГ [28][31]. В то же время в данной группе больных происходит нарушение сперматогенеза, что может быть вызвано резистентностью яичек к действию ФСГ или первичной дисфункцией данных органов [28][31]. Предполагается, что увеличение концентрации ФСГ нарушает восстановление сперматогенеза после трансплантации почки [31].

Женщины, страдающие тХБП, имеют повышенную концентрацию ФСГ и низкое соотношение гормонов ЛГ/ФСГ. Данные изменения вызваны как нарушением пульсирующего выброса ГнРГ, так и высокой концентрацией пролактина [31].

Снижение уровня эстрогенов и тестостерона, наблюдающееся у пациентов с ХБП, способствует ухудшению функции почек. Известно, что эстрогены играют важную роль в развитии вазодилатации почечных сосудов благодаря повышению выработки оксида азота (NO) [32]. Кроме того, показано, что в почках данные гормоны оказывают антиоксидантное действие, уменьшают процессы фиброза, а также ускоряют регенерацию клеточного эпителия. Также эстрогены принимают участие в регуляции гомеостаза фосфора в проксимальных почечных канальцах [33]. Прямое влияние тестостерона на функцию почек, и в частности на почечную гемодинамику, у людей до конца не изучено [34]. Опыты на животных показали, что высокие концентрации тестостерона могут повреждать подоциты [35]. В то же время низкие дозы тестостерона защищают почки от гипоксических изменений. Имеются доказательства наличия рецепторов данного гормона на афферентных артериолах клубочков почек и специфического дозозависимого эффекта тестостерона на тонус артериол в опытах на мышах [36]. Таким образом, изменения концентрации изучаемого вещества преимущественно влияют на внутриклубочковую гемодинамику [37].

В целом дисфункция гонадотропной оси является общей чертой пациентов, страдающих тХБП, вне зависимости от гендерной принадлежности. Гипоталамо-гипофизарно-гонадная ось при тХБП представлена на рисунке 2.

**Figure:**
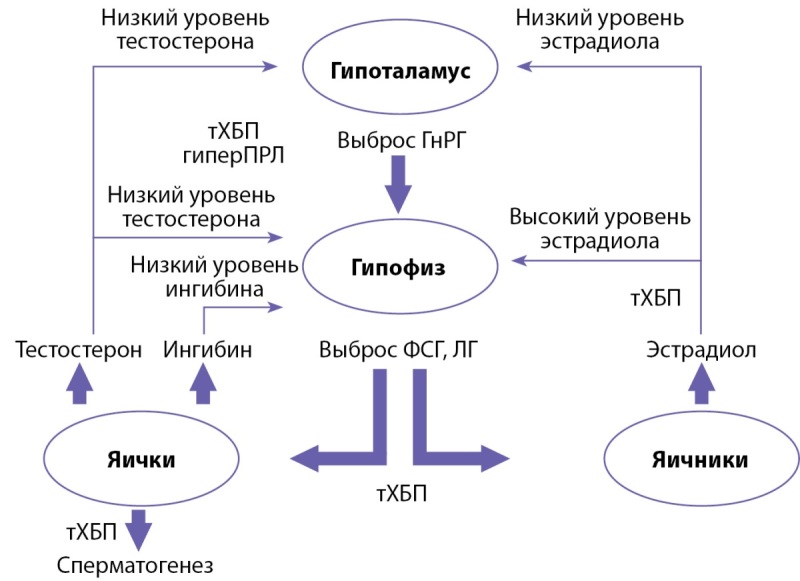
Рисунок 2. Гипоталамо-гипофизарно-гонадная ось при тХБП [адаптировано из 31]. Примечание: тХБП — терминальная стадия хронической болезни почек; ГнРГ — гонадотропин-рилизинг-гормон; ФСГ — фолликулостимулирующий гормон; ЛГ — лютеинизирующий гормон; гиперПРЛ — гиперпролактинемия.

Уменьшение концентрации эстрогенов и тестостерона отягощает течение ХБП и снижает качество жизни пациентов. Проведение диализной терапии в подавляющем большинстве случаев не приводит к нормализации концентрации гонадотропинов [25]. Применение заместительной гормональной терапии препаратами эстрогенов и тестостерона в данной группе больных ограничено в связи с отсутствием четких клинических рекомендаций, разработанных на основе ­результатов крупных рандомизированных исследований [28]. В настоящее время имеются данные, свидетельствующие о том, что пересадка почки способствует нормализации концентрации гонадотропинов у пациентов с тХБП. Так, спустя 6 мес после трансплантации почек происходит снижение уровней ЛГ и ФСГ [25][38], в то же время содержание тестостерона [39] и эстрогенов [38] может как повышаться, так и оставаться без существенных изменений [25][39].

Таким образом, тХБП характеризуется нарушением пульсирующей выработки ГнРГ и ЛГ, увеличением концентрации ФСГ и ЛГ, выраженными изменениями концентрации половых гормонов, при этом трансплантация почек не всегда приводит к их нормализации.

## ТИРЕОТРОПНЫЙ ГОРМОН

Тиреотропный гормон (ТТГ) — соединение, имеющее молекулярную массу 28–30 кДа и принадлежащее к семейству гликопротеиновых гормонов, так же, как ЛГ и ФСГ [40][41]. Тиреотропин-рилизинг-гормон (ТРГ) способствует высвобождению ТТГ в кровеносное русло. На поверхности клеток щитовидной железы ТТГ связывается со специфическими рецепторами, приводя к экспрессии генов, отвечающих за выработку трийодтиронина (Т3) и тетрайодтиронина (Т4) [41].

Влияние йодтиронинов на почки можно разделить на две группы: преренальные и прямые эффекты. Преренальные эффекты тиреоидных гормонов опосредованы изменением состояния сердечно-сосудистой системы, что оказывает влияние на почечный кровоток. В целом данные гормоны ускоряют почечный кровоток и снижают сопротивление почечных сосудов за счет как преренальных эффектов, так и прямого действия на почки [42]. Прямые эффекты йодтиронинов обусловлены регулированием процессов роста и функционирования почек. Под влиянием Т4 и Т3 изменяются секреция и реабсорбция электролитов. Гормоны щитовидной железы увеличивают активность Na-P (NaPi)-котранспортера, Na-H (NHE)-обменника, а также Na/K-АТФазы, усиливая реабсорбцию Na+ в проксимальных почечных канальцах. Йодтиронины влияют и на активность адренергических и дофаминергических рецепторов в клетках почечных канальцев, регулируют работу ренин-ангиотензин-альдостероновой системы [43].

В настоящее время известно, что не только нарушение синтеза йодтиронинов приводит к структурным и функциональным изменениям со стороны почек, но и ХБП оказывает влияние на работу органов оси гипоталамус-гипофиз-щитовидная железа [44].

Большинство пациентов, страдающих тХБП, имеют нормальную сывороточную концентрацию ТТГ, однако циркадный ритм с пиком выработки данного гормона поздним вечером и ранним утром нарушается, а также снижается ночное повышение и пульсаторное высвобождение ТТГ в кровеносное русло [28][41]. Наличие тХБП приводит к повышению гликозилирования молекулы ТТГ, что может удлинять период полувыведения данного вещества [41][43][44]. Метаболический ацидоз, развивающийся при уремии, нарушенный клиренс и увеличенный период выведения полураспада данного гормона способствуют развитию сниженной реакции тиреотрофов на ТРГ, вызывая нарушение высвобождения ТТГ в кровеносное русло [41][44].

Концентрация Т4 в сыворотке крови больных, имеющих тХБП, как правило, не изменена [28][41]. Однако, по данным ряда исследований, в группе пациентов с тХБП отмечаются низкий уровень общего Т4 и нормальная или незначительно сниженная концентрация свободного Т4, обусловленная нарушением связывания Т4 с транспортными белками плазмы крови [42]. При тХБП повышается активность фермента дейодиназы 3 типа с последующим образованием реверсивного Т3 (rT3). Однако в целом концентрация rT3 у пациентов с ХБП находится в пределах нормальных значений благодаря перемещению данного вещества из сосудистого русла во внутриклеточное пространство [41].

Содержание Т3 в плазме крови пациентов, страдающих тХБП, может снижаться или находиться в пределах референсных значений. Синдром низкого уровня Т3 является одним из наиболее ранних и распространенных нарушений функции щитовидной железы в данной группе пациентов [41][43][44]. Уменьшение концентрации Т3 в сыворотке крови происходит вследствие нарушения превращения Т4 в Т3, вызванного снижением активности фермента дейодиназы 1 типа. Кроме того, повышенный уровень воспалительных цитокинов (интерлейкина-6 (ИЛ-6) и фактора некроза опухоли-α) при тХБП способен ингибировать периферическую конверсию Т4 в T3 [41][43][44].

Нарушение функции почек влияет и на экскрецию йода с мочой. Уменьшение выделения йода почками приводит к его накоплению в щитовидной железе, вызывая длительный эффект Вольфа–Чайкова [43]. Изменения уровня гормонов оси гипоталамус-гипофиз-щитовидная железа при тХБП представлены на рисунке 3 [41].

**Figure:**
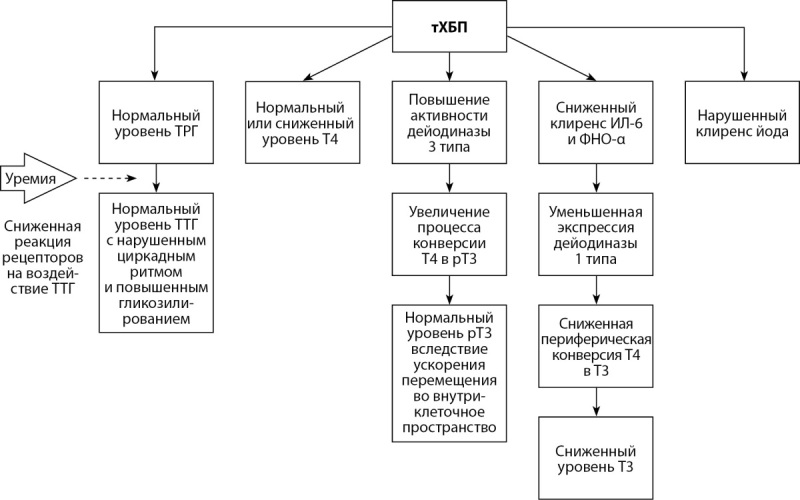
Рисунок 3. Ось гипоталамус-гипофиз-щитовидная железа при тХБП [адаптировано из 41].Примечание: тХБП — терминальная стадия хронической болезни почек; ТРГ — тиреотропин-рилизинг-гормон; ТТГ — тиреотропный гормон; Т4 — тироксин; Т3 — трийодтиронин; рТ3 — реверсивный Т3; ИЛ-6 — интерлейкин-6; ФНО-α — фактор некроза опухоли-α.

Описанные выше нарушения метаболизма ТТГ и йодтиронинов вызывают множество вопросов, касающихся интерпретации лабораторных данных и выбора тактики лечения пациентов, имеющих тХБП. Клиническое значение синдрома низкого уровня Т3 является спорным. Так, по данным Сarrero J.J. и соавт. (2007), сниженные концентрации общего Т3 у больных с тХБП коррелируют с более высокими уровнями маркеров воспаления (С-реактивным белком, ИЛ-6 и др.), недостаточностью питания, развитием эндотелиальной дисфункции, а также повышенной смертностью от любых причин, в том числе от сердечно-сосудистых заболеваний [45]. Результаты исследования Ozen K.P. и соавт. (2011) показали, что низкий уровень свободного Т3 связан с повышенной смертностью пациентов, получающих терапию гемодиализом [46]. В то же время Fernandez-Reyes M.J. и соавт. (2010) не выявили достоверной корреляции между содержанием свободного Т3 в плазме крови и показателями смертности пациентов с тХБП [47].

Также в настоящее время не разработана единая тактика лечения больных уремией, имеющих повышенный уровень ТТГ. Известно, что по мере снижения СКФ распространенность субклинического гипотиреоза неуклонно возрастает. По данным исследования Chonchol M. и соавт. (2008), около 18% пациентов с ХБП, не получающих лечение гемодиализом, наблюдаются с диагнозом «первичный субклинический гипотиреоз». Участники обследования с расчетной СКФ<60 мл/мин/1,73 м² имели большую вероятность повышения концентрации ТТГ по сравнению с участниками с расчетной СКФ≥60 мл/мин/1,73 м² после поправки на возраст, пол, уровень глюкозы в плазме натощак, концентрации общего холестерина и триглицеридов. Так, распространенность первичного субклинического гипотиреоза у пациентов с ХБП увеличивалась с 7 до 17,9% по мере снижения СКФ с ≥90 мл/мин до 60 мл/мин [48]. Тактика лечения субклинического гипотиреоза у больных, получающих лечение гемодиализом, может включать в себя ограничение потребления йода с пищей [41], а также терапию левотироксином натрия. Однако данное лечение не показало однозначного преимущества в отношении снижения смертности в изучаемой группе больных, что требует проведения дальнейших клинических исследований [44]. Нормализация уровня гормонов щитовидной железы происходит после трансплантации почки [25][43]. Так, исследование Kumar R. и соавт. (2014) продемонстрировало, что снижение концентрации ТТГ и свободного Т3 развивается к концу первого месяца после трансплантации почки [25].

В целом важно отметить, что нарушение концентрации ТТГ и йодтиронинов, наблюдаемое у пациентов с ХБП, не всегда следует рассматривать как показатель дисфункции работы щитовидной железы. Данные изменения могут являться отражением тяжелого хронического заболевания в качестве адаптации организма к катаболическим процессам [28].

## АДРЕНОКОРТИКОТРОПНЫЙ ГОРМОН

Адренокортикотропный гормон (АКТГ) — полипептид, состоящий из 39 аминокислотных остатков. Конечным продуктом оси гипоталамус-гипофиз-надпочечники является кортизол [14][49].

В физиологическом состоянии почки принимают участие в выведении кортизола и его метаболитов из организма благодаря превращению кортизола в кортизон 11b-гидроксистероиддегидрогеназой типа 2 (11-bHSD2). Данный фермент препятствует связыванию кортизола с минералокортикостероидными рецепторами в дистальных извитых канальцах и собирательных трубочках почек, таким образом предотвращая развитие гипернатриемии, гипокалиемии и гиперволемии. Глюкокортикоиды оказывают важное влияние на функцию почек вследствие повышения СКФ и изменения активности многочисленных ионных транспортеров, расположенных в канальцах почек [50]. По данным ряда исследований предполагается, что АКТГ вне зависимости от уровня кортизола способен оказывать нефропротективное действие благодаря уменьшению активности Т- и В-лимфоцитов. [51]. Также имеются данные о влиянии АКТГ на подоциты в клубочках почек. Подоциты содержат меланокортиновые рецепторы 1 типа (MCR1). Связывание АКТГ с MCR1 приводит к снижению активности ядерного фактора каппа β (NF-κβ), расположенного в клубочках почек, у пациентов с нефротическим синдромом. Таким образом, уменьшается количество поврежденных, нефункционирующих подоцитов вследствие воздействия на них NF-κβ [51].

При развитии ХБП изменяется метаболизм кортизола. Так, активность фермента 11-bHSD2 снижается, что приводит к повышению уровня данного гормона в сыворотке крови. Также нарушается связывание кортизола с транспортными белками. Уменьшаются процессы транспортировки данного гормона с помощью альбумина. В то же время связывание кортизола с транскортином остается неизменным. Концентрация АКТГ в данной группе больных находится в пределах нормальных значений или незначительно повышена [5]. Также отмечается сохранный суточный ритм секреции кортизола и АКТГ, что свидетельствует об отсутствии нарушения связи оси гипоталамус-гипофиз-надпочечники у пациентов с ХБП [5][25].

Проведение ЗПТ вызывает тенденцию к уменьшению концентрации АКТГ и кортизола, а также нормализации периода полувыведения кортизола по сравнению с группой пациентов, получающих консервативное лечение [5]. В то же время трансплантация почек снижает уровень кортизола к концу 1-го месяца после оперативного лечения. Однако через 6 мес возможно повторное повышение концентрации данного гормона параллельно с увеличением содержания креатинина в сыворотке крови. Интерпретация данных измерений может носить ошибочный характер в связи с тем, что все пациенты в исследовании, проведенном Kumar R. и соавт. (2014), получали глюкокортикостероиды до операции и находились на поддерживающей терапии после проведения трансплантации почек [13]. По данным Gracia-Iguacel С. и соавт. (2014), повышенная концентрация кортизола в сыворотке крови пациентов, получающих лечение гемодиализом, коррелирует с маркером воспаления (С-реактивным белком) и увеличивает риск смертности [52]. Таким образом, оценка содержания АКТГ и кортизола у пациентов с тХБП, получающих ЗПТ и после трансплантации почек, затруднена вследствие проведения терапии, оказывающей выраженное влияние на концентрацию данных гормонов в сыворотке крови.

В настоящее время имеются ограниченные данные по опыту применения АКТГ у пациентов, страдающих тХБП. По данным литературы, терапия АКТГ способствует улучшению функции почек при развитии рецидивирующего фокально-сегментарного гломерулосклероза трансплантата почки. Так, Anwar S. и соавт. (2015) показали, что применение АКТГ у пациента с гломерулосклерозом трансплантата почки в течение 4 нед приводит к стабилизации уровня креатинина и протеинурии и отмене сеансов гемодиализа [53].

Дальнейшие исследования помогут определить взаимосвязь между уровнем кортизола и АКТГ и прогнозом у пациентов с тХБП, а также оценить преимущества и недостатки применения препарата АКТГ у больных с трансплантатом почки.

## ВАЗОПРЕССИН

Вазопрессин представляет собой пептид, состоящий из 9 аминокислот [54]. В кровеносном руcле данное вещество связывается со специфическими рецепторами: V1a, V1b и V2. В почках преимущественно располагаются рецепторы V1a и V2. Рецепторы V1a обнаруживаются в междольковых артериях, нисходящем колене петли Генле, плотном пятне и собирательных трубочках почек. Активация данных рецепторов приводит к повышению уровня артериального давления за счет вазоконстрикции вследствие прямого действия вазопрессина на гладкомышечные клетки и опосредованного действия, вызванного увеличенной секрецией ренина. Рецепторы V2 локализованы в собирательных трубочках почек, плотном пятне и восходящем колене петли Генле и играют ключевую роль в процессах реабсорбции воды вследствие активации аквапоринов 2 типа, а также увеличивают процесс обратного захвата Na+ в собирательных трубочках [55]. Популяционное исследование PREVEND показало, что с помощью взаимодействия с рецепторами V2 вазопрессин участвует в экскреции альбумина с мочой, таким образом усиливая альбуминурию у пациентов с нормальной функцией почек [56].

В клинической практике определение концентрации вазопрессина затруднено, так как около 90% молекул данного вещества переносится тромбоцитами, остальные 10% представляют собой нестабильную фракцию, разрушающуюся в кровеносном русле [56]. Как правило, в клинических исследованиях оценивают уровень копептина, представляющего собой С-концевую часть предшественника вазопрессина [56]. Так, по данным Roussel R. и соавт. (2015), уровень копептина положительно коррелировал со степенью снижения функции почек и прогрессированием ХБП [57]. В то же время проведение сеансов гемодиализа у пациентов с тХБП не приводит к повышению концентрации вазопрессина и проявляется клинически снижением уровня артериального давления [58]. Особый интерес представляет определение концентрации копептина у пациентов после трансплантации почек. Так, Meijer E. и соавт. (2009) показали, что повышение уровня данного вещества коррелирует со снижением функции почечного трансплантата вне зависимости от исходной СКФ, возраста, пола и других известных факторов отторжения трансплантата [59]. Таким образом, в настоящее время появляется все больше данных, свидетельствующих о влиянии повышенной концентрации вазопрессина на прогрессирование ХБП.

В последние десятилетия разрабатывается новая группа препаратов — антагонисты рецепторов вазопрессина V1a или V2, которая, возможно, станет дополнительным средством для профилактики прогрессирования ХБП [56]. Дальнейшие исследования помогут выявить четкую связь между ХБП и вазопрессином.

## ЗАКЛЮЧЕНИЕ

ХБП — патологическое состояние, при котором по мере снижения функции почек отмечаются изменения содержания многочисленных гормонов, включая гормоны гипофиза. Данные нарушения становятся наиболее выраженными у пациентов с тХБП. Проведение трансплантации почек в большинстве случаев приводит к нормализации работы оси гипоталамус-гипофиз-периферические эндокринные железы. Изменения концентрации гормонов гипофиза у пациентов с тХБП суммированы в таблице.

**Table:** Таблица. Наиболее частые изменения концентрации гормонов гипофиза у пациентов с тХБП Примечание: тХБП — терминальная стадия хронической болезни почек; СТГ — соматотропный гормон; ИФР-1 — инсулиноподобный фактор роста-1; ЛГ — лютеинизирующий гормон; ФСГ — фолликулостимулирующий гормон; ТТГ — тиреотропный гормон; АКТГ — адренокортикотропный гормон.

Гормон	тХБП	ЗПТ
СТГ/ИФР-1	Нормальная, повышенная [8]	Нормальная, тенденция к снижению СТГ [5]
Пролактин	Повышенная [17][24]	Повышенная [18][22][24]
ЛГ	Повышенная, нарушение пульсирующей секреции [28–30]	Повышенная [25]
ФСГ	Повышенная [28][31]	Повышенная [25]
ТТГ	Нормальная с нарушением циркадного ритма, повышенная [28][41]	Нормальная, повышенная [48]
АКТГ	Нормальная, повышенная [5]	Тенденция к снижению [5]
Вазопрессин	Нормальная, повышенная [58]	Нормальная, тенденция к снижению [59]

По данным многочисленных исследований известно, что тХБП сопряжена с высоким риском смертности вследствие развития сложных патологических механизмов, которые могут усугубляться гормональными ­нарушениями. Изменения концентрации гормонов гипофиза при тХБП следует рассматривать как следствие снижения экскреторной, синтетической и регуляторной функций почек, однако данные нарушения отягощают функционирование организма и являются одними из наиболее важных проявлений уремического синдрома. Нормализация уровней гормонов у пациентов с тХБП, возможно, замедлит процесс снижения функции почек и приведет к уменьшению необходимости проведения ЗПТ и трансплантации почек, а также улучшит качество жизни данных больных. Совместная работа эндокринологов и нефрологов в диагностике гипофизарных нарушений и причин их развития с дальнейшей разработкой стратегии ведения пациентов может привести к снижению частоты осложнений и смертности пациентов с тХБП.

## ДОПОЛНИТЕЛЬНАЯ ИНФОРМАЦИЯ

Источник финансирования. Работа выполнена по инициативе авторов без привлечения финансирования.

Конфликт интересов. Авторы декларируют отсутствие явных и потенциальных конфликтов интересов, связанных с содержанием настоящей статьи.

Участие авторов. Маркова Т.Н. — проверка статьи, внесение коррективов, утверждение рукописи; Мищенко Н.К. — концепция и дизайн статьи, написание текста; Косова Е.В. — сбор и обработка материалов, написание текста. Все авторы одобрили финальную версию статьи перед публикацией, выразили согласие нести ответственность за все аспекты работы, подразумевающую надлежащее изучение и решение вопросов, связанных с точностью или добросовестностью любой части работы.
